# Impact of viral replication inhibition by entecavir on peripheral T lymphocyte subpopulations in chronic hepatitis B patients

**DOI:** 10.1186/1471-2334-8-123

**Published:** 2008-09-22

**Authors:** Jing You, Hutcha Sriplung, Alan Geater, Virasakdi Chongsuvivatwong, Lin Zhuang, Yun-Li Li, Hua Lei, Jun Liu, Hong-Ying Chen, Bao-Zhang Tang, Jun-Hua Huang

**Affiliations:** 1 Epidemiology Unit, Faculty of Medicine, Prince of Songkla University, Thailand; 2 Department of Infectious Diseases, The First Affiliated Hospital of Kunming Medical University, PR China; 3 Department of Hepatology, Third Municipal People's Hospital of Kunming, PR China; 4 Department of Infectious Diseases, Yunnan General Hospital of The Chinese People's Armed Police Forces, Kunming, PR China

## Abstract

**Background:**

To investigate dynamic fluctuations of serum viral load and peripheral T-lymphocyte subpopulations of chronic hepatitis B patients and their correlation during entecavir therapy.

**Methods:**

Fifty-five patients received entecavir 0.5 mg/d therapy. Serum HBV DNA load was measured by Real-Time-PCR, and the levels of peripheral T-lymphocyte subpopulations by flow cytometry biweekly, every four weeks and every eight weeks during weeks 1–12, 13–24 and 24–48, respectively. Multilevel modelling was used to analyse the relationship between these variables.

**Results:**

Of the 55 patients, all HBeAg positive and with detectable HBV DNA, the majority (81.8%) had serum levels of HBV DNA over 10^7 ^copies per milliliter. HBV viral load dropped sharply during the first two weeks. In 28 and 43 patients, the level became undetectable from week 24 and 48, respectively. Using pre-therapy level as the reference, a significant decrease in CD8^+ ^T cells and increase in CD4^+ ^T cells were found from week 12. Both parameters and CD4^+^/CD8^+ ^ratio steadily improved throughout the 48 weeks. Multilevel analyses showed that the level of decrement of HBV DNA was associated with the increment of T-lymphocyte activities only in the later period (4–48 week). After 4 weeks of therapy, for each log_10 _scale decrement of HBV DNA, the percentage of CD4^+ ^lymphocyte was increased by 0.49 and that of CD8^+ ^decreased by 0.51.

**Conclusion:**

T-lymphocyte subpopulations could be restored partially by entecavir treatment in patients with chronic hepatitis B concurrently with reduction of viremia.

## Background

Hepatitis B virus (HBV) infection remains an important health problem with more than 350 million chronically infected people worldwide, and approximately 1 million people die annually from HBV-related disease, such as liver failure, cirrhosis and hepatocellular carcinoma [1]. Infection with HBV in adults usually results in a self-limiting, acute hepatitis, which confers protective immunity and causes no further disease. In patients with an acute self-limiting HBV infection, specific CD4^+ ^and CD8^+ ^T cell responses are important for control of the infection [2]. Patients with a chronic HBV infection lack such vigorous, polyclonal, and multispecific T cell responses, but instead exhibit a weak, narrowly focused, or undetectable virus-specific T cell response [2-5]. The reasons for this lack of responsiveness and the mechanisms that contribute to the failure of the virus-specific T cell response in chronically infected patients are not understood [3-8]. Potential mechanisms include T-cell exhaustion, depletion, anergy, ignorance and T cell dysfunction and epitope mutation [6,7].

Most previous studies suggested that T cell failure is associated with a persistent high viral replication [9,10]. An efficient antiviral T cell response can be restored transiently by lamivudine treatment in patients with chronic hepatitis B (CHB) concurrently with reduction of viremia. This indicates the importance of viral load in the pathogenesis of T cell hyporesponsiveness in these patients [11-13]. However, the relationship between HBV specific T-cell response and HBV viral load in persistent infection is complicated by their close correlation and remains unclear [6,7]. Furthermore, correlation of T-cell failure with viral replication is likely to be a crucial step in the treatment of chronic hepatitis B [6,7]. Therefore, understanding of the immune response upon HBV infection and the mechanisms responsible for T-cell failure may help identify rational therapeutic strategies to restore T-cell failure, thus allowing successful stimulation of antiviral T cell responses for obtaining long-lasting viral suppression and disease remission. This may also offer us a possibility to better individualize HBV treatment based on the patient's immune condition and viral profile.

Two major types of antiviral drugs are being used for the treatment of chronic HBV: drugs that directly interfere with virus replication and drugs that modulate the HBV-specific immune response. Nucleoside and nucleotide analogues, such as lamivudine, adefovir and entecavir, directly inhibit reverse transcriptase and thereby impair viral replication. Interferon (IFN) has marked immunomodulatory, but less pronounced direct antiviral, effects. Recently, novel antiviral agents have been evaluated for their therapeutic potential for treatment of chronic hepatitis B [14-18]. Entecavir, one of the most promising of such agents, is a guanosine analogue and a potent inhibitor of the HBV polymerase. Recent studies suggested that over a 12-month treatment period the antiviral efficacy of entecavir would be superior to that of lamivudine [19-22]. It inhibits HBV DNA replication at three stages: priming of HBV DNA polymerase, a step involving the covalent linkage with guanosine triphosphate; the reverse transcription of the negative strand HBV DNA from the pregenomic messenger RNA; and the synthesis of the positive strand HBV DNA. The potency of entecavir for inhibiting HBV replication provides a possible avenue for documenting possible correlation between high viral load and T cell failure. It is important to know whether reduction of viral load resulting from antiviral treatment can cause a recovery of the impaired T cell response in CHB patients. The aim of this study was to analyze the dynamics of the peripheral T-lymphocyte subpopulations profile and viral load in HBeAg positive CHB patients during the first year antiviral therapy with entecavir.

## Methods

### Enrollment of study subjects

Fifty-five consecutive CHB patients (42 males and 13 females, age 16 to 60 years) who were recruited from the First Affiliated Hospital of Kunming Medical University, the Third Municipal People's Hospital of Kunming and the Yunnan General Hospital of The Chinese People's Armed Police Forces, between January 2006 and February 2007, were prospectively included into the study. All patients had presence of hepatitis B surface antigen (HBsAg) and HBeAg in the serum for at least 12 months; positive serum tests for HBV DNA with high viral load more than 10^5 ^copies/mL, documented on at least two occasions, at least 3 months apart, during the 12 months before entry; aminotransferase levels higher than 2 times the upper normal limit for at least 12 months; and liver biopsy taken within 12 months before enrollment showing chronic hepatitis [23]. Patients treated with immunosuppressive or antiviral therapy within 1 year before entry, and those with concurrent hepatitis C virus, hepatitis delta virus, human immunodeficiency virus infections, liver cirrhosis, causes of liver disease other than HBV, intravenous drug abuse, pregnancy, malignancy, chronic renal failure, or other serious medical illness that might interfere with this trial, were excluded. Informed consent was obtained from each study patient. The study protocol conformed to the guidelines of Declaration of Helsinki and was approved by ethics committees of the Faculty of Medicine of Prince of Songkla University and the First Affiliated Hospital of Kunming Medical University.

### Study design

This design used was single cohort with intervention study. The patients received entecavir at a daily dose of 0.5 mg for 48 weeks. Clinical, virological, biochemical and immunological parameters were assessed at baseline, then biweekly, every four weeks and every eight weeks during weeks 1–12, 13–24 and 24–48, respectively.

### Serological liver function tests and hepatitis B virus markers evaluation

Biochemical investigations including serum alanine aminotransferase (ALT), aspartate transaminase (AST) and total bilirubin (TBiL) were tested with routine automated techniques (upper limit of nomal; 40 U/L, 40 U/L and 17.1 μmol/mL, respectively) (AU2700, Japan). HBV markers (HBsAg, HBsAb, HBeAg, HBeAb, HBcAb, Anti-HBcAb IgM) were measured at a virological laboratory with the use of ELISA (enzyme-linked immunosorbent assay) method (Anthos 2010, Austria). The experimental methods followed the guideline written in the reagent kit (Sino-American Biotech Co., Ltd).

### Quantitative detection of HBV DNA

Serum HBV DNA load was assessed with real-time fluorescent quantitative polymerase chain reaction method (Real-Time-PCR) using a Lightcycler PCR system (FQD-33A, Bioer) with a lowest limit of detection of approximately 1000 viral genome copies/mL. The handling procedures were performed in strict accordance with the reagent kit (Shenzheng PG Biotech Co., Ltd.) package insert. The primer was provided in the kit, the reaction volume was 40 μL, and the reaction condition was 37°C for 5 min, 94°C for 1 min then 40 cycles as 95°C for 5 sec and 60°C for 30 sec.

### Peripheral blood T-lymphocyte subsets measurement

The blood samples were analysed with a Multi-Q-Prep processor (Coulter, USA) and thereafter with Epics-XL flow cytometry (FCM) (Coulter, USA). Lymphocytes were analysed using a gate set on forward scatter versus side scatter, and a three-color flow cytometry to combination reagent of CD3, CD4, CD8. Anti-human monoclonal antibodies CD3-PE-CY5/CD4-FITC/CD8-PE were from Immunotech, Ltd, USA. For each sample, the detection was analyzed with the CELLQuest software (Coulter, USA). The results were expressed as the percentages of CD3^+^, CD4^+ ^and CD8^+ ^T-cells found to be positive for the marker antigen in the total T cell population.

### Statistical analysis

Descriptive statistics were used to examine the age, sex, serum level of HBV viral load, T-lymphocyte subpopulation, HBeAg status, ALT, AST and total bilirubin. Continuous data were presented as means ± standard deviations and were compared by a two-tailed Student *t *test or one-way ANOVA adjusted for multiple comparision, as appropriate. Categorical data were compared by a two-tailed chi-square test or Fisher's exact test. The aim in longitudinal analysis was to investigate the effects of covariates both on the overall level of the responses and on changes of the responses over time. Thus, multilevel regression with random effects among subjects was employed to assess the independent effects of variables on peripheral blood T-lymphocytes. The changes of peripheral blood T-lymphocyte subpopulations during treatment are dependent variables of the first level nested within the subject which is at the second level. On the other hand, the effects of age, sex, treatment time (week) and the changes of viral load and HBeAg expression were treated as fixed effects common to all subjects. Variables yielding a P value of ≤ 0.2 in the univariate analysis were included in the multivariate analysis, and the model refined by backward elimination guided by the change in log likelihood of successive models. A final P value of less than 0.05 was considered statistically significant. Computations were carried out with the aid of R softwere version 2.6.0, and glmmPQL function [24]. The longitudinal plots were done with Epicalc package version 2.6.0.2 [25].

## Results

### Demographic characteristics and clinical features of CHB patients

Fifty-five consecutive patients were enrolled in this study. Characteristics of the patients are summarized in Table 1. The sample is predominated by male, full grown adults with a long period of HBV infection. The majority (81.8%) had serum levels of HBV DNA over 10^7 ^copies per milliliter. Most patients (73%, 40/55) were moderate chronic hepatitis with moderate activity and mild or moderate fibrosis (grade 3, stage 1–3; using the Scheuer grading and staging system) and 15% (8/55) patients were severe chronic hepatitis with marked activity and marked fibrosis and developing cirrhosis (grade 4, stage 2–4). No patients were with previous hepatic decompensation. No patient dropped out, and all completed the 48-week therapy.

**Table 1:** Characteristics of the CHB patients at entry to the study*

Characteristics	All patients (n = 55)
Sex (male/female)	42/13
Mean age (in years)^†^	30.9 ± 8.5
Duration of infection (years)^†^	13.8 ± 8.7
Serum ALT level (IU/L)^†^	251.4 ± 66.1
Serum AST level (IU/L)^†^	195.7 ± 81.6
Serum total bilirubin level (μmol/mL)^†^	26.7 ± 18.1
Serum HBV DNA (copies/mL)^‡^	
> 1.0 × 10^5 ^~ 1.0 × 10^7^	10 (18.2)
> 1.0 × 10^7^	45 (81.8)
HBV DNA (Log, copies/mL)^†^	7.9 ± 1.1
Diagnosis of chronic hepatitis (Histopathological results)^‡^	
Mild (grade 1–2, stage 0–2)	7 (12.7)
Moderate (grade 3, stage 1–3)	40 (72.7)
Severe (grade 4, stage 2–4)	8 (14.5)

* CHB, chronic hepatitis B; ALT, alanine aminotransferase; AST, aspartate aminotransferase; Normal values: ALT, ≤40 IU/L; AST, ≤40 IU/L; total bilirubin, ≤17.1 μmol/mL. † Date are expressed as mean ± SD; ‡ Data are expressed as no. (%).

### Changes in virological and enzyme parameters during the therapy

**Figure 1:**
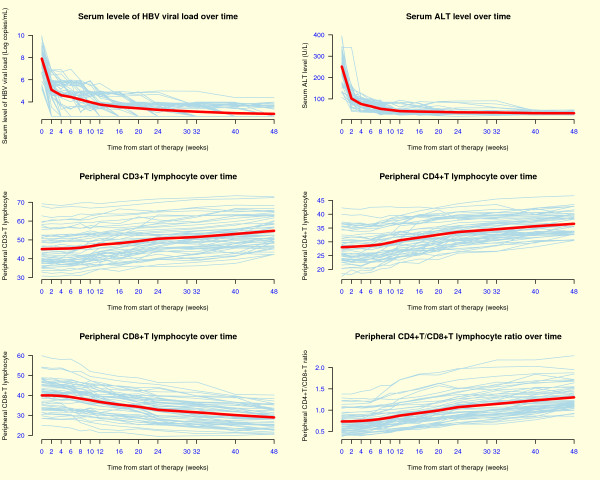
**Dynamic fluctuations of serum levels of viral load and ALT and peripheral T-lymphocyte subpopulations during entecavir treatment over time**. Note: The thick lines are the mean values.

HBV DNA levels declined sharply by around 3 log_10 _copies/mL during the first two weeks, with a highly significant reduction (p < 0.0001) at week 2 and thereafter, as compared to those at baseline (Table 2 and Fig. 1); 31%, 51% and 78% of the patients had undetectable serum HBV DNA levels at week 12, 24 and 48 respectively. The magnitude of HBV DNA reduction was up to 63% of the baseline level at the end of the study. The mean reduction in the serum HBV DNA levels at week 48 was 5 log_10 _copies per milliliter. At the end of week 24 and 48, the proportion of subjects losing HBeAg and converting to HBeAb positive were 6/55 (11%) and 9/55 (16%), and 4/55 (7.3%) and 9/55 (14.5%), respectively. Highly significantly decreasing serum aminotransferase and total bilirubin (ALT, AST and TBil) (p < 0.0001) occurred during the first 2 weeks of the study (Table 2 and Fig. 1). At week 48, ALT levels were normalized in 84% of the patients. At the end of 24^th ^and 48^th ^weeks, complete response (ALT normalization and HBV DNA and HBeAg loss) was observed in 11% and 15%, respectively. There was no evidence of drug resistance or adverse effect in CHB patients treated for up to 48 weeks.

**Table 2:** Response to treatment during entecavir therapy (n = 55) *

	Response to treatment over time (weeks)						
	
	pre (baseline)	2^nd^	4^th^	12^th^	16^th^	24^th^	48^th^
HBVDNA-negation^†^	0	8 (14.5)	13 (23.6)	17 (30.9)	21 (38.2)	28 (50.9)	43 (78.2)^g^
HBeAg-negation^†^	0	0	0	3 (5.5)	3 (5.5)	6 (10.9)	9 (16.4)^h^
HBeAg/HBeAb seroconversion^†^	0	0	0	1 (1.8)	2 (3.6)	4 (7.3)	8 (14.5)
ALT normalization^†^	0	0	7 (12.7)	31 (56.4)	39(70.9)	47 (85.5)	46 (83.6)
ALT normal/HBeAg & HBVDNA-negation^†^	0	0	0	3 (5.5)	3 (5.5)	6 (10.9)	8 (14.5)
HBV DNA (Log, copies/mL) ^‡^	7.9 ± 1.1	5.1 ± 1.21^d^	4.6 ± 1.3^d^	3.8 ± 0.9^d^	3.6 ± 0.8^d^	3.3 ± 0.7^d^	2.9 ± 0.5^df^
T-lymphocyte subpopulations (percentage) ^‡^							
CD3^+^T-lymphocyte	45.1 ± 9.0	45.3 ± 8.9	45.4 ± 8.9	47.4 ± 8.6	48.2 ± 8.7^a^	50.6 ± 8.4^c^	54.9 ± 7.9^df^
CD4^+^T-lymphocyte	28.1 ± 5.7	28.2 ± 5.5	28.4 ± 5.6	30.6 ± 5.1^b^	31.7 ± 5.4^c^	33.6 ± 4.6^d^	36.5 ± 3.7^dg^
CD8^+^T-lymphocyte	40.0 ± 6.7	39.9 ± 7.1	39.8 ± 7.0	36.7 ± 6.6^b^	35.4 ± 6.2^c^	32.8 ± 6.2^d^	28.9 ± 28.9^dg^
CD4^+^/CD8^+^T-lymphocyte ratio	0.7 ± 0.2	0.7 ± 0.2	0.8 ± 0.2	0.9 ± 0.3^b^	0.9 ± 0.3^d^	1.1 ± 0.3^d^	1.3 ± 0.3^dh^
Biochemichal measurements ^‡^							
Serum ALT level (IU/L)	251.4 ± 66.1	101.6 ± 43.4^d^	75.7 ± 23.4^d^	42.6 ± 15.8^d^	40.5 ± 15.0^d^	37.0 ± 13.32^d^	32.9 ± 6.5^df^
Serum AST level (IU/L)	195.7 ± 81.6	73.9 ± 62.3^d^	57.6 ± 18.9^d^	39.5 ± 14.0^d^	38.2 ± 14.5^d^	35.7 ± 8.1^d^	29.9 ± 4.7^dh^
Serum total bilirubin level (μmol/mL)	26.7 ± 18.1	11.0 ± 4.9^d^	10.9 ± 4.1^d^	10.7 ± 3.9^d^	10.9 ± 4.1^d^	10.3 ± 3.4^d^	9.6 ± 3.1^d^

*ALT, alanine aminotransferase; AST, aspartate aminotransferase; Normal values: ALT, ≤40 IU/L; AST, ≤40 IU/L; total bilirubin ≤17.1 μmol/mL; CD3^+^T, 61.1~77.0; CD4^+^T, 25.8~41.6; CD8^+^T, 18.1~29.6; CD4^+^/CD8^+^T ratio, 1.57~2.93.

† Date are expressed as no. (%);

‡ Data are expressed as mean ± SD.

^a^p < 0.05, ^b^p < 0.01, ^c^p < 0.001, ^d^p < 0.0001 *vs *pre;

^f^p < 0.01, ^g^p < 0.001, ^h^p < 0.0001 *vs *24^th^.

### Peripheral blood T-lymphocyte subpopulations changes

The effect of the entecavir therapy on alterations of the T cell subpopulations are shown in Table 2 and Figure 1. While the clearance of HBV DNA was relatively rapid, improvement of T-lymphocyte subpopulations was gradual but steady throughout the 48-week period. Only when viremia viral load level dropped to approximately 4 log_10 _copies/mL, at weeks 12–16, could significant increases in the CD3^+ ^cells and in the CD4^+^/CD8^+ ^ratio be observed (p < 0.05, p < 0.01, respectively). All T-lymphocyte parameters continued to improve and achieved highly significant difference from the baseline (p < 0.0001) by the 48th week. The increase in the CD4^+^/CD8^+ ^ratio was a combined result of an increase in the number of CD4^+ ^cells and a decrease in CD8^+ ^cells. The magnitude of increase in CD3^+^, CD4^+ ^cells and CD4^+^/CD8^+ ^ratio and decrease in CD8^+ ^cells was, respectively, 21.6%, 30.2%, 77.2% and 27.6% of pre-therapy levels at the end of study.

### Multilevel regression predicting peripheral blood T-lymphocyte subpopulations from relevant parameters

In Table 3, multilevel regression models using multivariate random coefficient analysis among subjects are separately summarized for CD3^+^, CD4^+ ^and CD8^+ ^cells, which are the dependent variables, respectively. The fixed effects part consists of coefficient (β) and standard error (SE) of the effect of each predicting variable which act equally to all subjects, while the random effects reflect variation of the baseline level outcome variable among the subjects. Interaction terms between log HBV DNA and period are significant for all T-lymphocyte parameters, indicating that effect of HBV DNA on these parameters after the first 2 week of therapy was significantly different from that in the initial period. The main effect of HBV DNA was not significant indicating no relationship during the initial period. The signs of the interaction term were negative for CD3^+ ^and CD4^+^, and positive for CD8^+ ^indicating that in the later period, the level of decrement of HBV DNA was associated with the increment of T-lymphocyte activities. These effects have already been adjusted for time or "week" as a continuous variable in the models. After 4 weeks of therapy, for each log_10 _scale decrement of HBV DNA, the percentage of CD4^+ ^lymphocyte was increased by 0.49 and that of CD8^+ ^decreased by 0.51. The relationship before week 4 was not statistically significant.

**Table 3:** Multivariate multilevel regression model predicting peripheral blood T lymphocyte subpopulations from relevant parameters (n = 55)*

	CD3^+^T Lymphocyte			CD4^+^T Lymphocyte			CD8^+^T Lymphocyte		
	
Variables	β	SE	P value	β	SE	P value	β	SE	P value
Fixed effects									
Intercept	44.50	1.31	--	28.09	0.88	--	39.85	1.09	--
Weeks **	0.21	0.01	<0.0001	0.18	0.01	<0.0001	-0.23	0.01	<0.0001
Period (4–48 week vs < 4 week)	0.99	0.75	0.19	1.92	0.72	<0.01	-2.07	0.86	0.02
HBV DNA (Log, copies/mL) **	0.07	0.09	0.47	-0.01	0.09	0.92	0.09	0.11	0.41
HBV DNA (Log, copies/mL) × period	-0.29	0.14	0.03	-0.48	0.13	<0.001	0.42	0.16	<0.01

Random effects:	Intercept = 8.49			Intercept = 4.71			Intercept = 6.02		
	SD = 1.73			SD = 1.66			SD = 1.97		

*β = Coefficients from the model, SE = Standard Error, SD = Standard Deviation. **Continuous variable.

## Discussion

This study demonstrated that entecavir results in an early rapid viral load reduction and continued virologic and biochemical improvement and HBeAg seroconversion through 48 weeks of treatment in CHB patients. Also a decreased viral load in serum (an inhibition of virus replication) during antiviral treatment was accompanied by a delayed restoration of peripheral T cell subpopulations. Although the relationship was not significant during the initial period (≤ 2 week), the level of decrement of HBV DNA was associated with an increment of T-lymphocyte activities in the later period (4–48 week). These responses were sustained up to the end of the observation period.

Our data showed that entecavir treatment led to a rapid drop of viremia in all patients and a slower HBeAg/HBeAb seroconversion. Most patients experienced improvements in serum ALT values after initiation of entecavir therapy. The efficacy of entecavir appears to result from its potent suppression of HBV replication, and this rapid viral suppression occurred especially during the first two weeks. The virologic and biochemical efficacy of entecavir continued through 48 weeks. The improvement of virologic and biochemical parameters found in this study was in agreement with recent studies of patients with chronic hepatitis B [19-22]. Global phase III studies demonstrated that after one year of treatment, entecavir is superior to lamivudine [19-22] in its ability to reduce HBV DNA to undetectable levels, normalize ALT, and improve liver histology in nucleoside-naive HBeAg-positive and -negative patients and lamivudine-retractory HBeAg-positive patients. Direct comparison of entecavir and adefovir for a duration of 24 wk showed a decline of HBV DNA of 6.97 log_10 _copies/mL for entecavir and 4.84 log_10 _copies/mL for adefovir. PCR undetectability (HBV DNA < 300 copies/mL) was reached in 45% of entecavir treated patients *vs *13% of those receiving adefovir [26]. Significant difference in rapid viral load reduction between entecavir and adefovir was as early as day 10 (-0.655, 95% CI: -1.30, -0.01) [26]. A similar pattern was also observed between entecavir and lamivudine [19-22]. The virologic and biochemical efficacy of entecavir is maintained with prolonged treatment for up to 96 weeks [27].

To our knowledge, no data have been reported concerning the synchronous effects of entecavir on the serial measurement of peripheral blood lymphocyte subpopulations and serum HBV DNA concentrations in patients with chronic hepatitis B. During entecavir-therapy, we detected a shift in the CD4^+^/CD8^+ ^ratio, owing to a relative decrease in the number of CD8^+ ^cells and increase in the number of CD4^+ ^lymphocytes, and an increase in the number of CD3^+ ^cells, concomitantly with a quantitative reduction in viral replication. The benefit of entecavir on T cell profile was not immediately significant at the time of rapid reduction of viral load during the first 2 weeks. T cell subpopulations restoration had a tendency to increase or accumulate gradually after initiation of treatment. Although the relationship was not significant during the initial period (≤ 2 week), the level of decrement of HBV DNA was associated with the increment of T-lymphocyte activities in the later period (4–48 week). Our results also showed that the magnitude of restoration of these parameters was maintained up to the end of the study. The independent effect of viral load on peripheral T-lymphocyte subpopulations profile found in this study was partly in accordance with previous studies. Stoop *et al *[28] and Lau *et al *[29] reported that inhibition of viral replication induced by adefovir therapy resulted in a increased HBV-specific proliferation and IFN-γ production, and a transient increase in HBV-specific CD4^+ ^T-cells, but no increase in HBV-specific CD8^+ ^T-cells. Kondo *et al *[30] suggested that lamivudine therapy induced mainly cytotoxic T lymphocytes (CTLs) that were less frequent before the therapy. Since recovered CTLs maintained the ability to produce interferon-gamma in response to peptides, these CTLs apparently contribute to the efficacy of lamivudine therapy in patients with hepatitis B. It was reported by Boni *et al *[11-13] that an efficient antiviral T cell response including HBV-specific CD4^+ ^and CD8^+ ^cells can be restored by lamivudine treatment in CHB patients concurrently with reduction of viremia, but the restoration of HBV-specific T-cell reactivity is only transient. In their study, after an initial increase of frequency of functionally efficient T cells (first 5 months of therapy), peripheral blood CD4^+ ^responses declined to pretreatment levels and this degree of reactivity was maintained after therapy discontinuation, throughout the follow-up period. In addition, this transient restoration of HBV-specific T-cell was also observed by Mizukoshi *et al *[31], who suggested that decreased T cell responsiveness during prolonged therapy was associated with increased prevalence of lamivudine-resistant HBV mutants and increased HBV titers. So, possible reasons for persistant restoration of T-cell function observed in our study are that entecavir in the treatment of CHB patients has a stronger antiviral activity specific for HBV and that resistance to entecavir is lower than that to lamivudine [19-22].

Our results reveal that T cell failure was significantly associated with viral replication level. The stronger independent predictive effect of time (week) on T cell improvement might be explained as the effect of treatment course. As a matter of fact, the restoration of peripheral T cell profile observed in our study is attributed to the effect of treatment with entecavir, not time (week) itself. However, entecavir reacts by directly inhibiting HBV DNA replication, not by modulating the HBV-specific immune response [19-22]. Indeed, our data showed that the significant changes of T-cell profile in CHB patients occurred when viremia viral load level dropped to around 4 log_10 _copies/mL and thereafter during entecavir-therapy. Furthermore, the decline of viremia viral load was clearly related with time (week). Therefore, these observations indicate that the restoration of impaired T cell profile may be actually directly related with the reduction of viral load resulting from inhibition of viral replication. Taken together with recent reports of previous studies [11-13,28-31], all this accumulating evidence that efficient antiviral T cell response can be restored by mono-antiviral treatment in CHB patients concurrently with reduction of viremia demonstrates the importance of viral load in the pathegenesis of T cell failure in chronic HBV infection.

This study suggests that entecavir can effectively reduce HBV viral load resulting from inhibition of virus replication and also maintain a low level for at least 48 weeks. As a consequence, an increase of CD3^+ ^and CD4^+ ^T cells, accompanied with a decrease of CD8^+ ^lymphocytes and a restoration of CD4^+^/CD8^+ ^ratio to a near normal profile are evident and also maintained for the whole study period. As the antiviral action of entecavir is directly on the virus, with no known direct effect on T cell subpopulations [19-22], our results are most likely explained by a dose-response causal relationship between viral load and peripheral T-lymphocyte subpopulations. Thus a high viral load may be one important factor that contributes to T cell failure and the latter could be restored by inhibiting virus replication through efficient antiviral treatment.

The strength of this study lies in the large sample size and the measurements of T-lymphocyte subpopulations using modern advanced flow cytometry technology and viral load with quantitative Real-Time-PCR method. A limitation of this study is that the specific T-lymphocyte subpopulations and liver-derived T-lymphocytes as well as their functionality were not explored concurrently. Another one is that long-term effect of entecavir was not known from this data set. Although the strong correlation between viral load and T-lymphocyte subpopulations is illustrated, further studies are needed to confirm the T cell responsiveness during prolonged therapy and after withdrawal of entecavir, and to elucidate the complex interactions among them.

Our results describe a dynamic relationship between T cell subpopulations and serum viral load that could potentially be used for a better design of HBV treatment in chronic hepatitis B patients. Inhibition of viral replication with agents such as novel nucleotide analogues may enhance the likelihood that therapeutic stimulation of the antiviral T cell reaponses will induce long-lasting viral suppression and HBV antigen seroconversion, ultimately leading to recovery from disease. Further clinical studies are needed to explore this possibility in persistent HBV-infected patients.

## Conclusion

In this study, profound effects on cellular distribution and viral replication were found during entecavir-therapy. The inhibition of virus replication induced by treatment with entecavir results in a partial restoration and improvement of the impaired cellular immune response. The restoration was clearly associated with the decreases in serum viral load, indicating a close correlation between viral load and T-lymphocyte subpopulations.

## Abbreviations

ALT: alanine aminotransferase; AST: aspartate transaminase; HBV: hepatitis B virus; HBV DNA: hepatitis B virus DNA; HBsAg: hepatitis B surface antigen; HBeAg: hepatitis B envelope antigen; CHB: chronic hepatitis B; ELISA: enzyme-linked immunosorbent assay; Real-Time-PCR: real-time fluorescent quantitative polymerase chain reaction.

## Competing interests

The authors declare that they have no competing interests.

## Authors' contributions

JY, HS, AG and VC conceptualized the study. JY and staff of the research group assisted with the data collection. JY was responsible for data management and data analysis. JY, HS, AG and VC were responsible for interpretation of data. HS, AG, VC and LZ provided advice and review. JY, HS, AG and VC collaboratively wrote the manuscript. All authors read and approved the final manuscript.

## Pre-publication history

The pre-publication history for this paper can be accessed here:


